# The Amino Acid Arginine 210 of the Response Regulator HrpG of *Xanthomonas citri* subsp. *citri* Is Required for HrpG Function in Virulence

**DOI:** 10.1371/journal.pone.0125516

**Published:** 2015-05-11

**Authors:** Florencia A. Ficarra, Cecilia G. Garofalo, Natalia Gottig, Jorgelina Ottado

**Affiliations:** Instituto de Biología Molecular y Celular de Rosario, Consejo Nacional de Investigaciones Científicas y Técnicas (IBR-CONICET), Ocampo y Esmeralda, Rosario, 2000, Argentina; Virginia Tech, UNITED STATES

## Abstract

*Xanthomonas citri* subsp. *citri* colonizes its hosts through the trafficking of effector proteins to the plant cell by the type III protein secretion system. In *X*. *citri* subsp. *citri*, as in other plant pathogens, the *hrp* cluster encodes the type III protein secretion system and is regulated by the transcription factors HrpG and HrpX. HrpG belongs to the OmpR family’s response regulator of EnvZ/OmpR two-component signal transduction system. Here, we show that the arginine 210 residue is crucial for the transcriptional activity of HrpG revealed by the absence of disease in host plants and hypersensitive response in non-host plants when a strain carrying this point mutation is used in plant infiltration assays. Also, this strain showed decreased expression levels of *hrp* genes in bacteria grown in culture or when they were recovered from citrus leaves. Moreover, we show for the first time that HrpG binds to both *hrpX* and its own promoter, and the change of the arginine 210 by a cysteine does not prevent the binding to both promoters. Nevertheless, *in vitro hrpX* transcription was observed only with HrpG whereas no transcription was detected with the R210C mutant. HrpG was able to interact with itself as well as with the mutant R210C suggesting that it functions as a dimer. The mutant protein R210C showed altered protease sensitivity, suggesting that Arg210 is essential for protein active conformation and thus for transcriptional activity. Our results indicate that arginine 210 in HrpG, as it may occur with this conserved residue in other members of this family of response regulators, is not required for DNA binding whereas is essential for *hrp* genes transcription and therefore for pathogenicity and HR induction.

## Introduction

Citrus canker caused by the bacterium *Xanthomonas citri* subsp. *citri* (Xcc), affects citrus production worldwide, mainly by the potential threat that constitutes infected material to citrus canker-free areas and the market restriction imposed in international trade. This represents the major economic impact of the disease, in addition to economic losses due to defoliation, twig dieback, premature fruit drop and general debilitation of the tree [1]. Xcc enters host plant tissues through stomata or wounds and then colonizes the apoplast of leaves, stems and fruits, causing cell hypertrophy and hyperplasia that leads to typical raised corky lesions [2].

Xcc colonizes citrus plants through the secretion of virulence effector proteins by the type III protein secretion system (T3SS) [3–5]. This system is encoded by the *hrp* [for hypersensitive response (HR) and pathogenicity] cluster, which translocates effector proteins into the plant cell and is indispensable for pathogenicity in the host plant and for the induction of HR in non-host plants. The *hrp* cluster consists of six operons (*hrpA* to *hrpF*) harboring more than 20 different genes that are classified as: *hrp*, only found in phytopathogens; *hrc* (*hrp* conserved in plant and animal bacterial pathogens), which constitute the core of the translocon; and *hpa* (*hrp* associated), which contribute to pathogenicity and to HR induction in non-host plants, but are not essential for bacterial pathogenic interactions with plants (for a review, see [6]).

In Xanthomonas, the expression of *hrp* genes is regulated by two transcription factors: HrpG and HrpX. HrpG belongs to the OmpR family’s response regulator of EnvZ/OmpR two-component signal transduction system that regulates expression of the porin genes *ompF* and *ompC* in response to changes in the osmolarity of the growth medium in *Escherichia coli* [7]. OmpR is a winged helix-turn-helix DNA-binding protein composed by two domains, an N-terminal receiver or phosphorylation domain and a C-terminal DNA binding domain. The structure of the C-terminal domain of *E*. *coli* OmpR has been solved and it has three α-helices flanked on two sides by antiparallel β-sheets, an N-terminal four-stranded β-sheets and a C-terminal hairpin that interacts with a short β-strand connecting helices α1 and α2 to form a three-stranded β-sheet. The topology of this domain is β1- β2- β3- β4- α1- β5- α2- α3- β6- β7 [8, 9]. Further, amino acids important for DNA recognition by OmpR and activation of the transcription of *ompC* and *ompF* have been identified. By means of NMR studies of the DNA binding domain of OmpR, residues that change their chemical shifts upon DNA binding were identified and phenotypic analysis confirmed their roles in activating transcription. It has been proposed that OmpR, first binds to its high affinity site as a monomer making contacts with thymine and guanine bases via Val203 and Arg207 and with the phosphate backbone via Arg209 in the recognition helix α3, and a second OmpR molecule binds subsequently [10].

Recently, a putative cognate sensor kinase for HrpG named HpaS was identified in *Xanthomonas campestris* pv. *campestris*. Several experimental evidences put forward that HpaS is responsible for HrpG phosphorylation since mutation of *hpaS* almost completely abolished the HR induction and virulence. Further, both proteins physically interact in bacterial two-hybrid and protein pull-down assays, and a mutant in *hpaS* showed reduced phosphorylation of HrpG *in vivo*. However, *in vitro* phosphotransfer from HpaS to HrpG could not be demonstrated [11]. Moreover, the post-transcriptional regulator RsmA is able to bind to the 5’ untranslated region of *hrpG* and by stabilizing *hrpG* mRNA leads to increased accumulation of HrpG protein [12].

HrpG regulates negatively its own expression while activates the expression of *hrpX* [13]. HrpX is an AraC-type transcription factor that activates the six *hrp* operons as well as type III secretion system effector genes in Xcc [14] and *X*. *campestris* pv. *campestris* [15], while in the deeply studied *Xanthomonas campestris* pv. *vesicatoria* HrpG also controls the expression of *hrcC* [16]. Several HrpX-regulated genes have a consensus sequence motif: the plant-inducible promoter (PIP) box (TTCGC-N15-TTCGC), in their promoter regions [17] and it has been demonstrated that HrpX binds to the PIP box [18]. Nevertheless, genes without a PIP box or with an imperfect one have been found to be regulated by HrpX [19, 20]. In Xcc, a genome-wide microarray analysis was carried out to characterize the HrpG and HrpX regulons and revealed that 232 and 181 genes are regulated by HrpG and HrpX, respectively. Among them, expected genes such as type III secretion system genes, type III secretion system effector genes and type II secretion system substrate genes were identified. Furthermore, genes involved in cellular activities such as amino acid biosynthesis, oxidative phosphorylation, pentose-phosphate pathway, transport of sugar, iron and potassium; and phenolic catabolism were also found to be regulated by HrpX and HrpG, The fact that HrpG regulates the expression of other regulatory genes indicates that HrpG is a Xcc master regulator involved in the development of the disease [14]. However, there is no direct evidence about the ability of HrpG to recognize and bind to the promoter sequences of the genes it regulates.

In this study we demonstrate that HrpG binds to both *hrpX* and its own promoter and that the residue arginine 210, entirely conserved in this family of transcription regulators that belongs to the DNA recognition α3 helix, is not required for DNA binding whereas is essential for *hrp* genes transcription and therefore for pathogenicity and HR induction.

## Results

### The residue Arg210 of HrpG is essential for pathogenicity and HR induction

**Fig 1:**
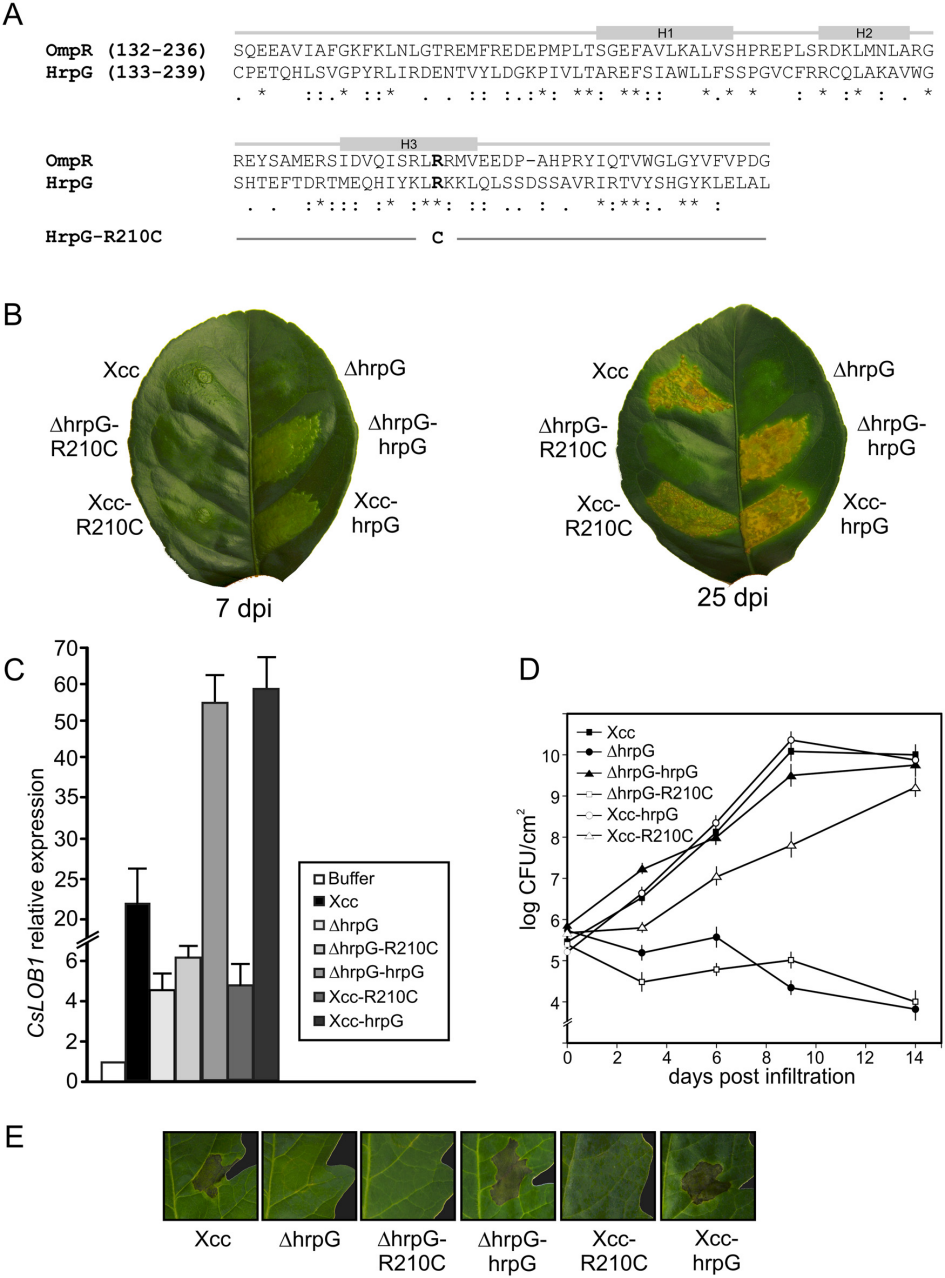
Characterization of the residue Arg210 of HrpG in Xcc pathogenicity and HR induction. (A) Sequence alignment and secondary structure assignments of a region of the DNA-binding domain of OmpR from *E*. *coli* and HrpG from Xcc. Helices α1, α2 and α3 are depicted with grey boxes. Asterisks (*) indicate identical residues, colons (:) are conservative replacements and full stops (.) are semiconservative replacements. Arg209 in OmpR sequence and Arg210 in Xcc sequence are depicted in bold. This residue turns into Cys210 in HrpG-R210C sequence (B) Xcc wild type, deletion mutant ΔhrpG, complemented strains ΔhrpG-R210C and ΔhrpG-HrpG and Xcc carrying the wild type and R210C mutant copy, Xcc-HrpG and Xcc-R210C, respectively, were inoculated at 10^7^ CFU/ml in 10 mM MgCl_2_ into the intercellular spaces of fully expanded citrus leaves. Representative leaves are shown 7 dpi (left panel) and 25 dpi (right panel). (C) RT-qPCR to determine *CsLOB1* expression levels in leaves after 24 hours of inoculation with Xcc strains. Bars indicate the expression levels relative to buffer infiltrations. Values are the means of four biological replicates with three technical replicates each. (D) Bacterial growth of the Xcc strains in citrus leaves. Values represent the mean of three samples from three different plants. Error bars indicate standard deviations. (E) Xcc variants were inoculated at 10^7^ CFU/ml in 10 mM MgCl_2_ in tomato leaves and a representative photograph after 24 h is shown.

In Xcc, as well as in other Xanthomonas, *hrp* gene expression is induced *in planta* and in the XVM2 medium, a minimal medium that mimics the plant apoplastic environment, while it is not expressed in rich medium [21]. To achieve *hrp* gene expression in non-inducing media, a Xcc strain with additional copies of *hrpG* (Xcc-hrpG) was obtained by cloning this gene in the pBBRMCS-3 expression vector, since it has been previously observed that this strategy increases *hrp* gene expression [22]. Several transconjugant strains were analyzed in pathogenicity tests and they caused typical canker symptoms in citrus leaves although these symptoms appeared earlier in infections with Xcc-hrpG strains than with the wild type bacteria (Fig 1B). Interestingly, a Xcc-hrpG strain that was less virulent at the first days of the pathogenic process was detected. The sequencing of the *hrpG* copy carried in this strain revealed a conversion of cytosine 628 to thymine caused by the PCR reaction during the cloning procedure. This was reflected in a change of the arginine 210 to cysteine in this HrpG mutant protein that was named HrpG-R210C (Fig 1A). Arg210 belongs to the C-terminal domain of the protein and it is entirely conserved among all the OmpR family members [23]. This mutation in *hrpG* exerted a dominant effect in view of the delay in the appearance of symptoms besides the presence of the genomic *hrpG* copy and this mutant strain was named Xcc-R210C. Typical water-soaking symptoms appeared at 7 days post infiltration (dpi) with Xcc while only a mild chlorosis and a slight reaction in the infiltration site was observed for Xcc-R210C at this time (Fig 1B, left leaf). At 25 dpi, lesions produced by Xcc-R210C were similar to Xcc lesions albeit with slightly less necrotic areas (Fig 1B, right leaf).

To further evaluate the contribution of Arg210 to HrpG function we constructed a Xcc *hrpG* deletion mutant by marker exchange mutagenesis. Similarly to the results previously observed using another Xcc *hrpG* deletion mutant that lost its pathogenicity in grapefruit [14], ΔhrpG showed no disease symptoms in orange leaves (Fig 1B). As expected, this strain could be complemented with a plasmid carrying the wild type copy of *hrpG* (ΔhrpG-hrpG) but not with the mutant R210C (ΔhrpG-R210C). As occurred with Xcc-hrpG, the presence of a constitutive wild type *hrpG* in the mutant strain ΔhrpG accelerated the development of symptoms in comparison with the wild type strain (Fig 1B).

In order to further evaluate the differences observed in virulence among these strains we analyzed *CsLOB1* expression by a RT-qPCR assay. This gene belongs to the Lateral Organ Boundaries (LOB) gene family of transcription factors and its expression is induced by the Xcc effector protein PthA4 [24, 25]. Accordingly, a mutant in the T3SS that is not able to cause disease does not induce the expression of *CsLOB1* gene [26]. Consistent with the pathogenicity test results, in leaves infected with Xcc, Xcc-hrpG or ΔhrpG-hrpG an induction of *CsLOB1* was observed at 1 dpi, with major levels of induction in infections with strains that carry the *hrpG* gene in a plasmid. In leaves inoculated with ΔhrpG, ΔhrpG-R210C or Xcc-R210C a slight induction of the expression of this gene was observed, similar to the results obtained with the T3SS mutant [26], supporting the fact that Xcc-R210C is delayed in the development of lesions (Fig 1C). To further characterize the behavior of the different strains, their growth in citrus leaves was quantified. The results showed that ΔhrpG and ΔhrpG-R210C did not grow while Xcc-hrpG and ΔhrpG-hrpG grew similarly to Xcc wild type, and Xcc-R210C grew slower at the first 10 days and then reached the population size of Xcc (Fig 1D). In view of the possibility that Xcc-R210C may be loosing the plasmids during the assay, we quantified bacteria extracted from plant tissues at different times in media supplemented with appropriate antibiotics and observed no loss of plasmids even 30 dpi (data not shown).

To assess the ability to elicit HR in non-host plants, bacterial suspensions of the different strains were infiltrated in tomato leaves. Xcc-hrpG and ΔhrpG-hrpG as well as Xcc were able to induce HR while inoculations with ΔhrpG, ΔhrpG-R210C and Xcc-R210C did not display HR symptoms (Fig 1E). The fact that the latter strain could not elicit HR, even in the presence of the genomic wild type *hrpG* copy, supports the idea that the mutation is exerting a dominant negative effect.

### Arg210 is essential for HrpG-mediated transcriptional activation

**Fig 2:**
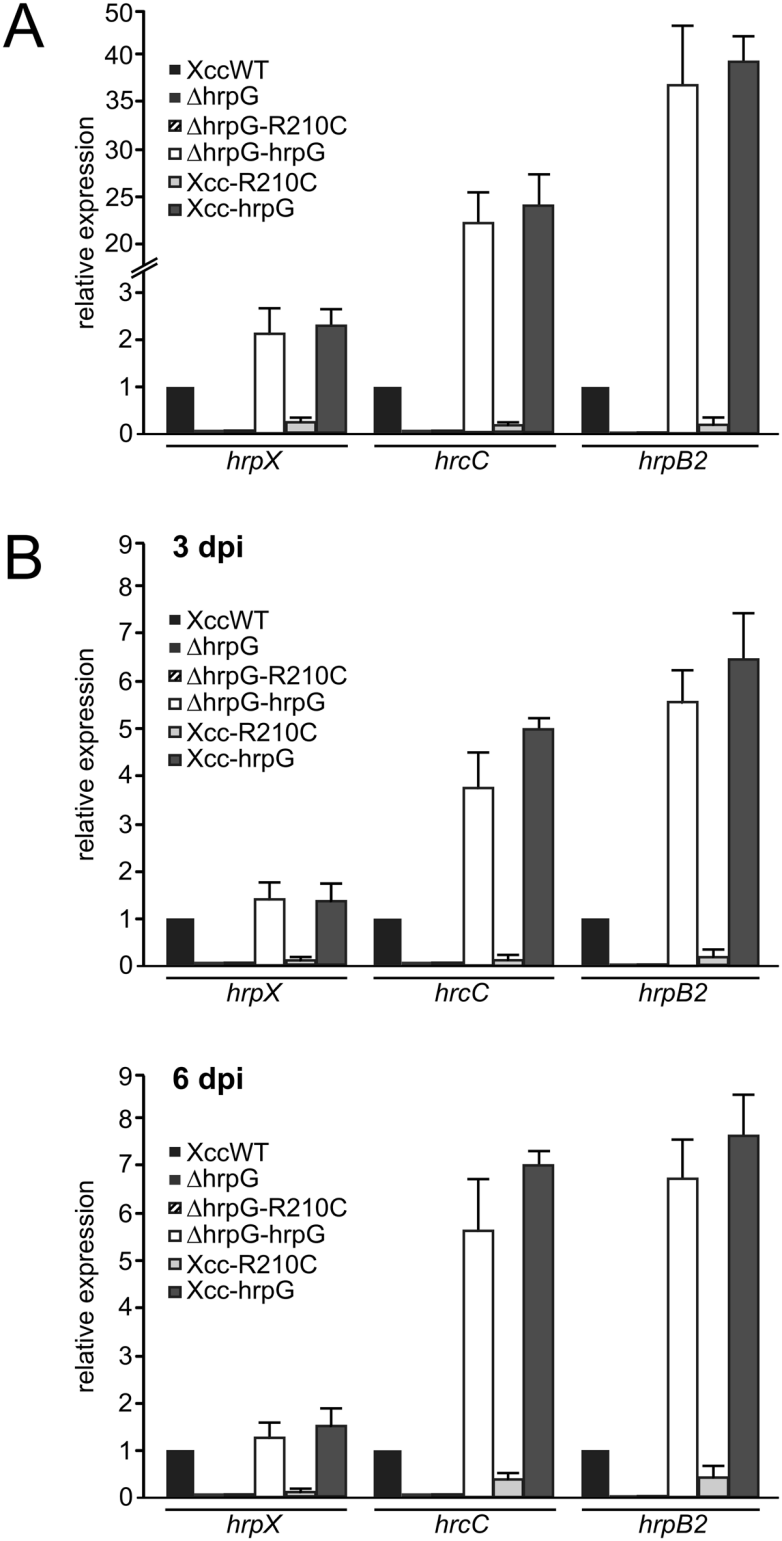
Expression of T3SS genes up-regulated by HrpG depends on the wild type copy of *hrpG*. (A) qRT-PCR of *hrpX*, *hrcC* and *hrpB2* of total RNA obtained from Xcc, deletion mutant ΔhrpG, ΔhrpG-R210C, ΔhrpG-HrpG, Xcc-R210C and Xcc-HrpG strains grown in XVM2 were assayed. (B) As in (A) but RNA was obtained from bacteria recovered from infiltrated tissue at 3 and 6 dpi before RNA extraction. As a reference gene, a fragment of *rpoB* gene was amplified. Values represent the means of four independent experiments. Error bars indicate standard deviations. Data were statistically analyzed using one-way ANOVA (p<0.05).

In order to evaluate the capacity of the mutant HrpG-R210C to activate transcription we analyzed the expression of *hrpX* and *hrcC*, both reported to be directly activated by HrpG in *X*. *campestris* pv. *vesicatoria* [27] and *hrpB2*, activated by HrpX [14]. To this end, the different strains were grown for 16 h in XVM2 medium and transcript levels of these genes were analyzed by qRT-PCR assays. As expected, Xcc-hrpG and ΔhrpG-hrpG showed an increase in *hrp* genes expression compared to Xcc wild type while ΔhrpG and ΔhrpG-R210C strains could not active transcription of these genes (Fig 2A). Interestingly the expression of HrpG-R210C in a wild type background lowered *hrp* genes expression levels more than four times compared to Xcc, confirming the results obtained in plant-pathogen assays (Fig 2A). Having established that Arg210 is required for HrpG-mediated gene expression, we evaluated *hrpX*, *hrcC* and *hrpB2* expression in plant-pathogen interactions by qRT-PCR assays. RNA was obtained from bacteria recovered from *C*. *sinensis* infected leaves at 3 and 6 dpi. A similar expression pattern to that obtained for bacteria cultured in XVM2 was observed with expression levels higher for 6 than for 3 dpi. Once again, the presence of the variant R210C reduced the levels of *hrp* genes transcripts while the wild type HrpG enhanced them (Fig 2B). The levels of transcripts in Xcc-R210C, even if at both times were lower than the ones in Xcc wild type, were more than two times higher (p<0.05) for *hrcC* and *hrpB2* at 6 dpi than at 3 dpi, in agreement with the results that lesions caused by Xcc-R210C equals those caused by the wild type strain later in the infection process (Fig 1B, right leaf). In general these results evidenced that Arg210 is essential for HrpG to induce the transcriptional activation of *hrp* genes and a mutation in this residue renders the bacteria less infective in host plants and impairs the ability to trigger the HR in non-host plants.

### Arg210 is not essential for HrpG binding to the *hrpX* and *hrpG* promoters

**Fig 3:**
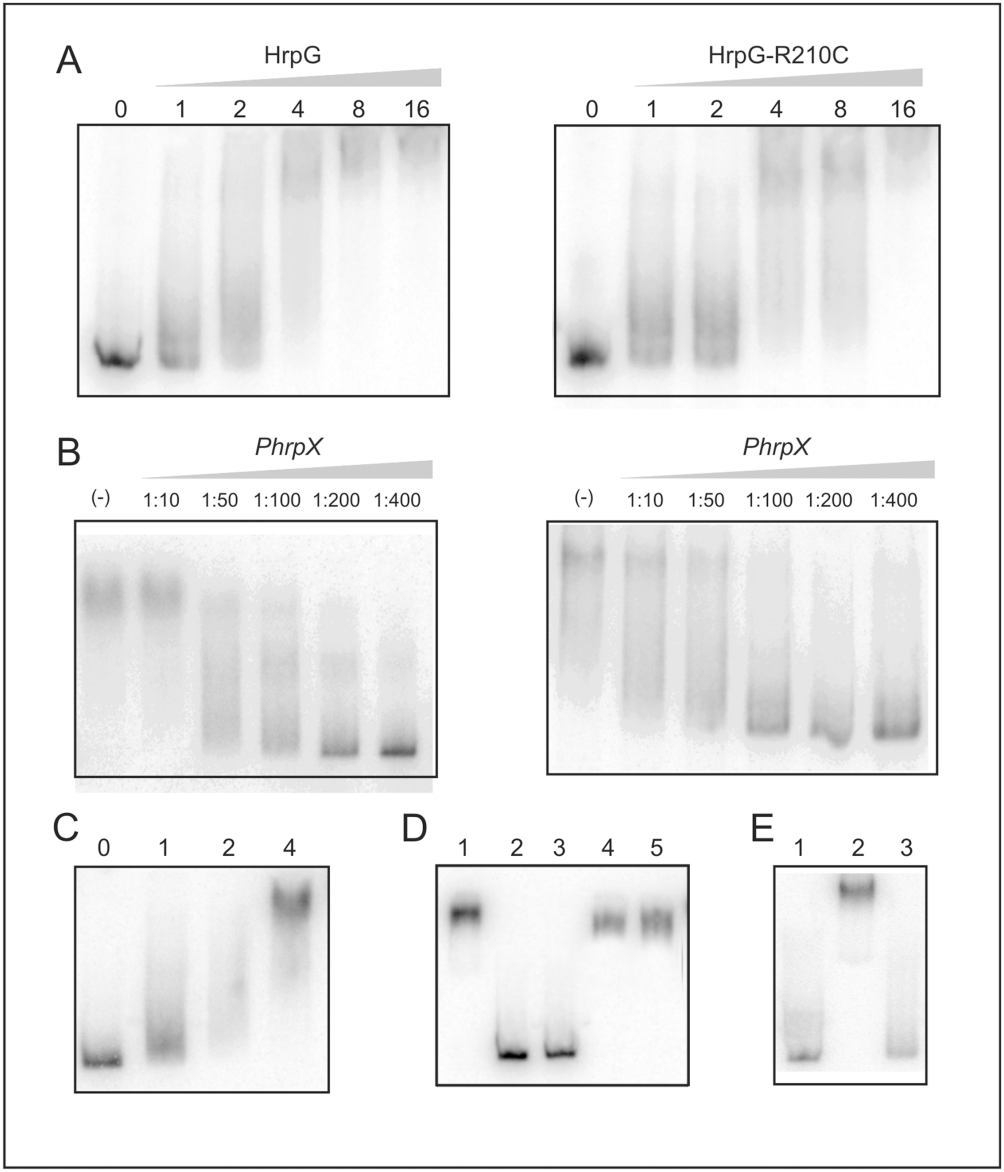
A substitution of Arg210 by cysteine does not prevent binding to DNA promoter regions. (A) Electrophoretic mobility shift assay of ^32^P-labeled *hrpX* promoter and purified HrpG and HrpG-R210C. Numbers in the top of the lanes indicate pmoles of protein added to the assay. (B) Complex competition assays with excess unlabeled DNA: Lanes labeled (-) show the complex between HrpG (left panel) and HrpG-R210C (right panel) with 8 pmol of protein. Competition was performed using the ratio indicated in each lane. (C) EMSA of ^32^P-labeled *hrpX* promoter and a mixture 1:1 of HrpG and HrpG-R210C with the total pmol indicated on top of each lane. (D) Control EMSA of ^32^P-labeled *hrpX* promoter and purified Trx in the same conditions as HrpG to discard unspecific interactions. Lane 1 shows the binding of 16 pmoles of HrpG to P_hrpX_ as control. Lanes 2 and 3: P_hrpX_ was incubated with 15 and 30 pmoles of Trx, respectively in the same conditions as for HrpG. Lanes 4 and 5: unspecific competition assay in which the HrpG-P_hrpX_ complex was challenged with 200 ng of poly-dIdC (lane 4) or salmon sperm DNA (lane 5). (E) Lane 1: free probe, lane 2: binding of 16 pmoles of HrpG to P_hrpX_ as control, lane 3: P_hrpX_ was incubated with 25 μg of a protein extract obtained from *E*. *coli* bearing the pET32 empty vector.

Considering that HrpG-R210C did not activate *hrp* genes transcription, we wondered whether Arg210 is required for HrpG binding to DNA. Initially, we evaluated the HrpG binding capacity to a 444-bp DNA fragment encompassing the region between -414 to +30 of the *hrpX* promoter (P_hrpX_) by EMSA and we observed a mobility shift in the presence of the protein (Fig 3A). Then, we assayed the binding of the mutant HrpG-R210C to P_hrpX_ and like the wild type protein, HrpG-R210C was able to bind to this promoter sequence with a similar affinity as the wild type protein (Fig 3B), suggesting that Arg210 is not necessary for the binding of HrpG to this promoter. Next, competition experiments were performed. Initially, protein-DNA complexes using 8 pmoles of HrpG or HrpG-R210C, quantity at which protein-DNA complexes were completely formed, were competed with an excess of unlabeled DNA probe. The results showed that competition was similar for the complex formed by either protein, putting forward that in both cases the binding is specific and with a similar affinity (Fig 3B). To further evaluate whether the both proteins have similar binding capacity to the promoter, the labeled DNA probe was incubated with a mixture (1:1) of wild type HrpG and HrpG-R210C to analyze the effect of the heterodimer on DNA binding. Once more, similar band shifts to the observed with any of the two proteins separately were observed (Fig 3C). Several controls were performed for these experiments. Since we could not obtain a purified soluble HrpG or HrpG-R210C, both proteins were expressed and purified fused to Trx and these recombinant proteins were assayed in the binding experiment described above. Therefore the same binding assays were performed with Trx and compared with the fused HrpG protein (Fig 3D, lane 1). As expected Trx at 15 and 30 pmoles, purified from an *E*. *coli* strain carrying the pET32 empty vector was not able to form a complex with P_hrpX_ (Fig 3D, lanes 2 and 3). Also, challenging of the HrpG- P_hrpX_ complex with poly-dIdC and salmon sperm was done and these DNAs could not compete with P_hrpX_ discarding unspecific interactions (Fig 3D, lanes 4 and 5, respectively). Moreover, the labeled P_hrpX_ was incubated with an *E*. *coli* protein extract obtained from a strain bearing the pET32 empty vector and no complex was observed (Fig 3E lane 3). Then, as HrpG is able to regulate negatively its own expression (S1 Fig) [13], we evaluated if it is able to bind to its own promoter. So, the binding and competition assays were performed with both proteins and the P_hrpG_ promoter and similar results to the ones observed for P_hrpX_ were obtained (S2 Fig). Overall, these results show that even though Arg210 is required for transcriptional regulation, it is not involved in DNA binding in the conditions assayed.

These results demonstrated that Arg210 is essential for transcriptional activation whereas dispensable for DNA binding, therefore we hypothesized that the lack of transcriptional activation may be due to an impairment in the interaction of HrpG-R210C with the α-subunit of the RNA polymerase (αCTD), as it has been proposed in the case of OmpR [8]. To study this, the Xcc αCTD polypeptide was expressed and purified and binding assays were performed to evaluate if this polypeptide causes a supershift of the complexes of bound HrpG and HrpG-R210C to the promoter regions. We observed that for both complexes the presence of αCTD did not change the mobility (S3 Fig), suggesting that in the conditions tested HrpG does not interact with αCTD or that this latter is not responsible for the activation of transcription.

### Arg210 is required for HrpG to activate *hrpX in vitro* transcription

**Fig 4:**
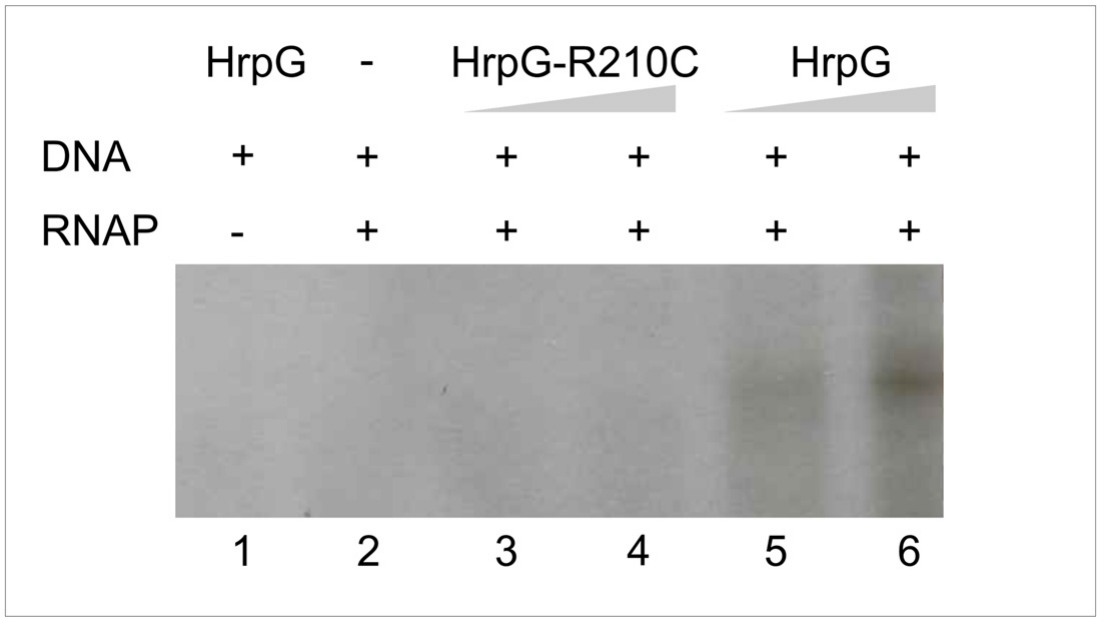
HrpG-R210C is unable to activate transcription from HrpX promoter. RNA was generated *in vitro* from a PCR product template containing the promoter as well as 200 bp downstream of the putative transcription start site of the corresponding gene. For *in vitro* transcription, templates were incubated with 16 (lane 3) and 32 pmol (lane 4) of HrpG-R210C protein or 16 (lane 5) and 32 (lane 6) of HrpG protein before the start of transcription by addition of 1 U of RNAP and NTPs. Negative controls were set up without addition of RNAP (lane 1) or HrpG (lane 2).

To further analyze whether the HrpG Arg210 is actually required to activate *hrpX* transcription, *in vitro* transcription assays were performed. A PCR product that includes *hrpX* promoter region as well as 200 pb downstream from the annotated transcription start site was obtained. The product was incubated with pure HrpG wild-type or HrpG-R210C, and *E*. *coli* RNA polymerase and [α-^32^P] UTP to detect nascent RNA. HrpG was able to initiate transcription evidenced by a ≈ 200 bp band that corresponds to the expected fragment. Moreover, a more abundant transcript product was observed when the amount of HrpG was raised. On the contrary, no bands were detected when the incubation was performed with the variant R210C. As controls the DNA fragment was incubated only with the transcription factor or the polymerase and in both cases no transcript was detected (Fig 4). These results give support to the expression analysis previously performed (Fig 2) and reinforce the requirement of HrpG Arg210 to activate transcription.

### HrpG could interact with itself and with the mutant variant HrpG-R210C

**Fig 5:**
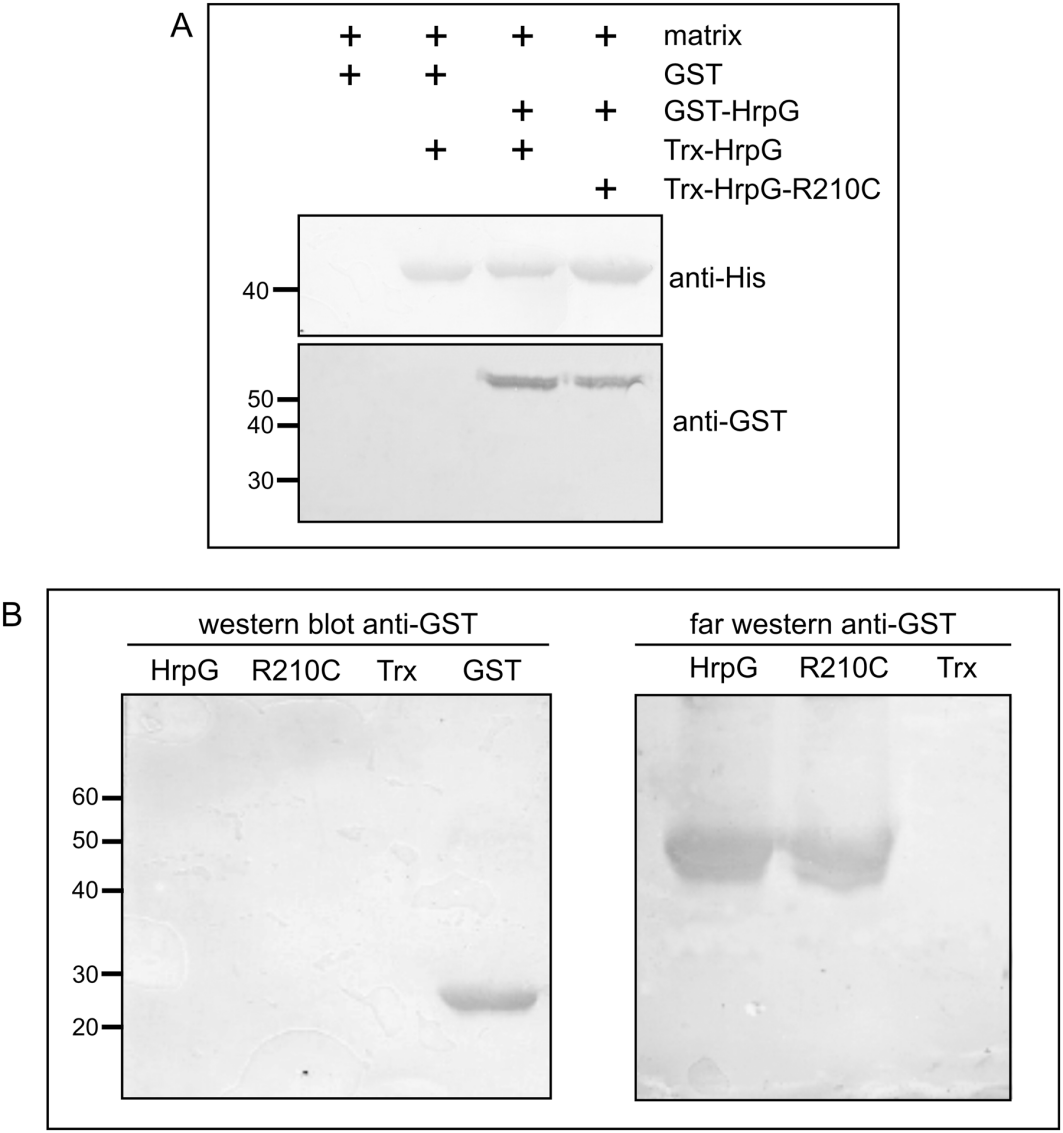
HrpG interacts with itself and with HrpG-R210C. (A) Pull-down assays showing *in vitro* interaction studies with HrpG fused to GST (GST-HrpG) and HrpG and HrpG-R210C fused to thioredoxin. Proteins eluted from the matrix were analyzed by immunoblotting using anti-His and anti-GST antibodies. (B) Western and Far-Western blots showing interactions between HrpG and HrpG and HrpG-R210C fused to thioredoxin. The Western blot was incubated with anti-GST (left panel) and in the Far-Western blot (right panel) the nitrocellulose membranes were overlayed with 50 μg of GST-HrpG and after washing, probed with anti-GST antibody.

Previously it has been observed that HrpG is able to interact with itself in yeast two-hybrid assays putting forward that HrpG may be able to dimerize [28]. In this regard and taking into account our results: (i) that HrpG-R210C is dominant in a wild type background, (ii) that it does not activate transcription, (iii) that it has similar affinity for *hrpG* and *hrpX* promoters than the wild type protein, we hypothesized that HrpG-R210C may be able to dimerize with HrpG and in that manner sequester wild type protein in inactive heterodimers. Therefore, we performed some experiments to analyze HrpG-HrpG-R210C interaction. A pull-down assay was done in which either HrpG or HrpG-R210C was incubated with the matrix and then GST-HrpG was added. After protein elution, a SDS-PAGE was run and results showed that GST-HrpG was present in the eluate of HrpG and HrpG-R210C but did not elute with the matrix alone, suggesting that interactions are specific (Fig 5A). Also, a Far-Western blot assay was used to confirm the interaction between HrpG and the mutant protein. Fig 5B shows that HrpG interacts with itself as well as with HrpG-R210C.

### HrpG and the mutant HrpG-R210C display different resistance to limited proteolysis

**Fig 6:**
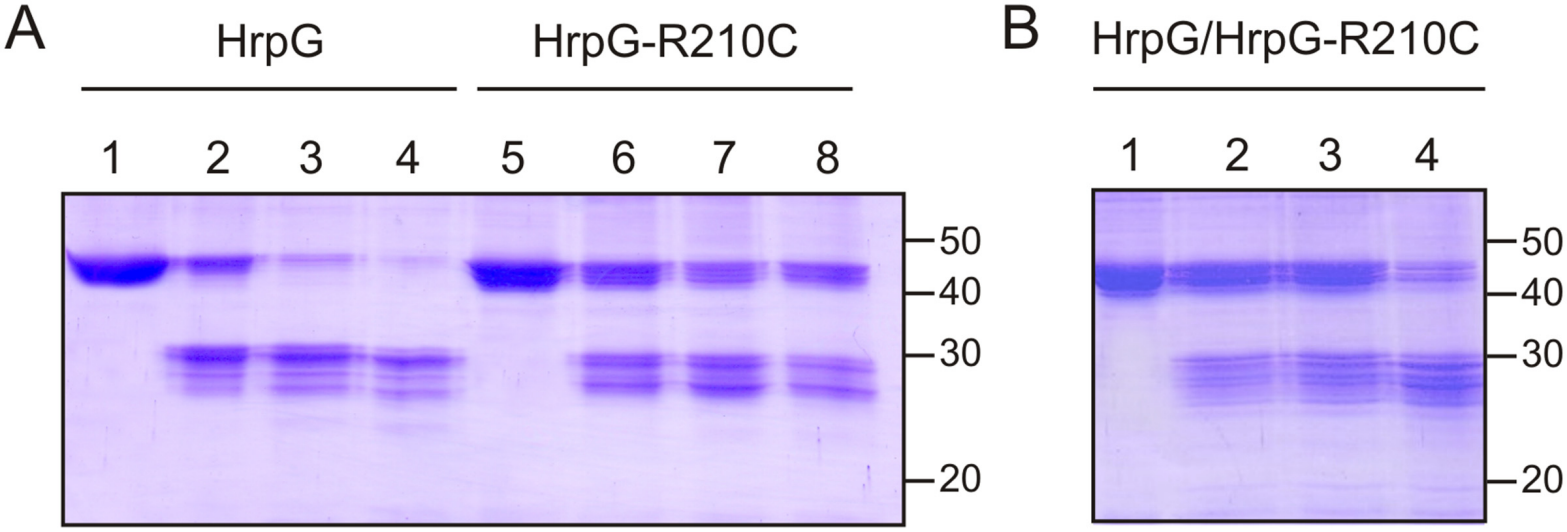
Resistance to limited proteolysis of the two protein variants. **(A)** HrpG and HrpG-R210C were subjected to trypsin proteolysis for 30 min at room temperature. Lanes 1 and 5 are the undigested samples (5 μg), lanes 2 and 6, proteins were digested with 1:250, lanes 3 and 7 with 1:125 and lanes 4 and 8 with 1:62.5 (w/w) trypsin. (B) A mixture 1:1 of HrpG and HrpG-R210C was subjected to trypsin proteolysis for 30 min at room temperature. Lanes 1 is the undigested sample (5 μg), in lanes 2–4 proteins were digested with 1:250, 1:125 and 1:62.5 (w/w) trypsin, respectively.

With the aim to identify conformational differences between HrpG and HrpG-R210C, a limited proteolysis assay with trypsin was performed. HrpG and HrpG-R210C resistance to increasing concentrations of protease was assessed after 30 min of incubation (Fig 6A). At the lowest concentration used, the fusion proteins were digested showing a band of approximately 29 kDa that corresponds to HrpG after the digestion of the fused Trx. Notably, for HrpG a major band of 29 kDa was observed while for HrpG-R210C two major bands of approximately 29 and 27 kDa were present, suggesting that the presence of the mutation alters the conformation of the C-terminal domain of the protein that becomes more accessible to the protease. The band corresponding to Trx-HrpG-R210C purified protein (Fig 6A, lane 5) is already split into two bands with eletrophoretic mobilities differing in approximately 2 kDa, suggesting that the mutation renders the protein more susceptible to natural proteolysis. Even if it did not affect the binding to DNA, it may be crucial for transcriptional activity. Moreover, Trx-HrpG was digested at lower trypsin concentrations than the variant R210C as almost no fusion protein was present when incubation was performed with the protease:protein highest ratio used (Fig 6A, lane 4). In the case of HrpG-R210C, the fusion protein remained, though with less intensity even at this highest protease condition, suggesting a different conformation (Fig 6A, lane 8). Then, we assessed the protease sensitivity of a mixture (1:1) of wild type HrpG and HrpG-R210C and an intermediate pattern between the ones observed with each protein was obtained (Fig 6B).

## Discussion

T3SS is essential for Xcc pathogenicity allowing the translocation of effector proteins directly into plant cells to interfere with plant defense mechanisms and in this way the bacteria can multiply and cause disease. In this context, HrpG as a protein that regulates the expression of the *hrp* cluster encoding the T3SS is essential for this process, and it was previously demonstrated that a Xcc mutant lacking *hrpG* is not pathogenic [14]. In this work we obtained a point mutant in *hrpG* that makes the Xcc strain that carries this copy non-virulent due to the presence of a simple amino acid substitution in the C-terminal region of HrpG. Here, we showed that in Xcc-R210C, in which the mutant variant is encoded in a replicative plasmid, the wild type genomic copy of *hrpG* is not able to counteract the effect of the mutated HrpG in bacterial virulence, at least at the first days of infection. This hypothesis is supported by the lack of complementation of the mutant strain ΔhrpG with the expression of the plasmid-encoded variant R210C. Similarly, a change of Arg to Cys at position 220 in a plasmid-encoded OmpR in a wild type background also exhibited dominance and it has been suggested that the loss of function phenotype may be caused by the interaction between wild-type and mutant polypeptides, the latter overproduced due to the multicopy state [29]. Moreover, Xcc-R210C could not elicit an HR in non-host plants, suggesting that even if a lower expression of *hrp* genes in this strain allows the T3SS assembly during host plants infection, it is not sufficient to elicit the rapid plant defense response that occurs during HR and in which the bacteria is not growing inside the non-host plant tissue [3]. In this context, the observation that *CsLOB1* expression, which reveals that PthA4 effector is translocated into the host cell, is lowered in citrus leaves infected with Xcc-R210C supports the idea that the T3SS is not completely functional in this strain in the initial stages of the infection process. Also, we showed that HrpG and HrpG-R210C are able to dimerize and that with similar affinity they bind to both *hrpX* and *hrpG* promoter regions. This suggests that, despite having different amino acid residues, both protein structures allow the correct interaction with regulatory DNA sequences. However, the presence of Cys210 in the mutant protein may affect critical interactions with other amino acids that may be crucial for the activation of gene transcription and in fact we observed that HrpG-R210C was not able to activate *hrpX* transcription. Further, the mutant protein showed altered protease sensitivity, suggesting that Arg210 is essential for protein active conformation. Overall, these results suggest that there is a competition between both proteins for the binding to DNA and when together they probably acquired a non-functional heterodimer conformation that is not able to activate transcription.

**Fig 7:**
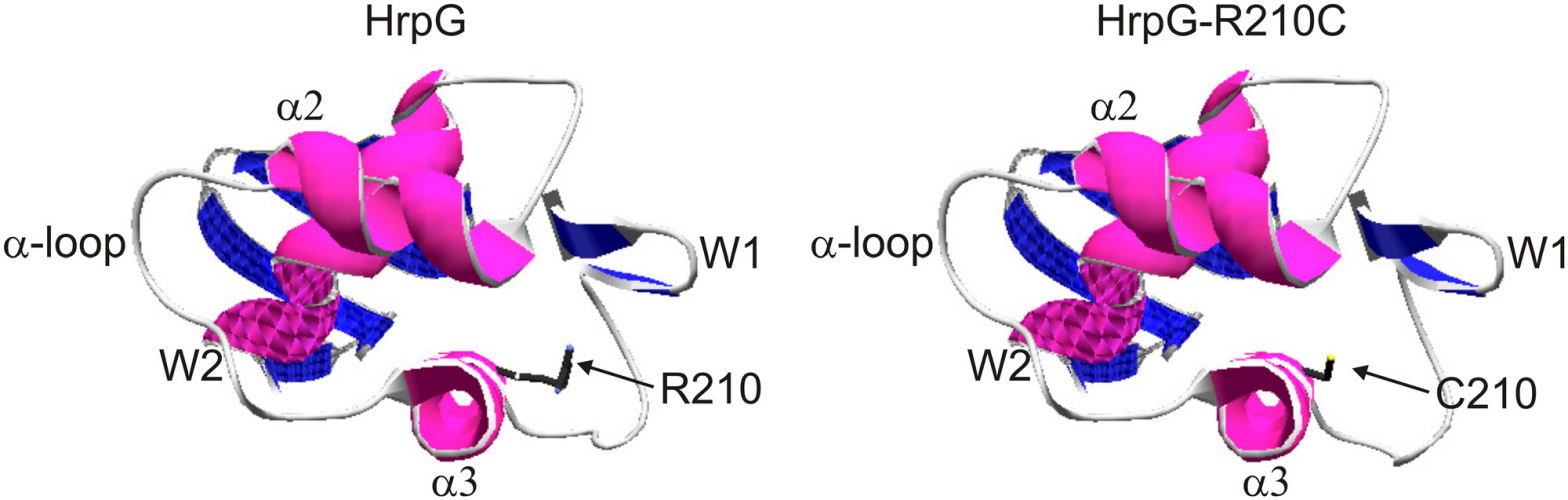
Three-dimensional structure models of HrpG and HrpG-R210C C-terminal domains. Modeling was done with the SwissModel-SPDViewer program based on the structure of OmpR from *E*. *coli*. Both structures are shown in a spatial orientation similar to that adopted by OmpR in its interaction with DNA [10]. α-helices are shown in pink while β-sheets in blue. In black the side chains of the amino acids arginine (R210) and cysteine (C210) are shown. W1 and W2: indicate wings 1 and 2, respectively; and α-helices 2 and 3 and the α-loop are also indicated.

Previously, with the aim to obtain *X*. *axononopodis* pv. *vesicatoria* HrpG mutants that confer constitutive expression of *hrp* genes in rich medium, several HrpG point mutations were obtained by random mutagenesis. The mutants obtained E44K, H194R and D199N could activate the transcription of *hrp* genes in rich medium [30]. On the other hand, in *E*. *coli* some mutations present in the C-terminus of OmpR led to loss of function [10, 31, 32]. It has been proposed that this region in OmpR might interact with the α-subunit of RNA polymerase and modulate its activity [23] and even though we did not observed any direct interaction between HrpG and the CTD of the α-subunit of RNA polymerase, we may approximate to HrpG understanding by comparing it with the family member OmpR which structure is known. The Xcc HrpG R210C mutation is located in a region of OmpR that belongs to the α3 helix and the α2-loop-α3 region forms a helix-turn-helix motif. While helix α3 is proposed to be the recognition helix that interacts with the major groove of DNA, α2 helix corresponds to the positioning helix [23] and it has been proposed that Arg209 (homologous to Arg210 of HrpG) contacts the DNA phosphatidic backbone whereas residues Val203 and Arg207 would contact with thymine and guanine bases in the recognition sequence, respectively [10]. In fact some differences between OmpR and HrpG may exist since in the latter the presence of Arg210 is not critical for DNA binding at least in the conditions assayed. Modeling HrpG and HrpG-R210C C-terminal region structure with the one from OmpR revealed that the overall structure of a wHTH motif is conserved in the mutant (Fig 7), suggesting that the binding should not be affected, consistent with the binding results. However, the importance of the Arg209 in the OmpR structure is quite significant since it may establish interactions with other amino acids that may be critical to activate gene transcription. In OmpR, wing 1 is defined by strands β5 and β6 and is attached to the recognition helix α3 through hydrogen bonds formed between residues Arg209 and residues Ile222 and Thr224. Indeed these residues are highly conserved among the members of this family of regulator proteins [23]. Nevertheless, their role in transcription activation such as binding to subunits of the RNA polymerase remains to be demonstrated.

In conclusion, our work shows for the first time the binding of HrpG to *hrpX* and to its own promoter and gives the first example of a point mutation with loss of function for Xcc HrpG. This substitution does not change the affinity of DNA binding compared with wild type HrpG protein but is crucial for *hrp* genes transcription activation and for the pathogenicity of the bacteria responsible for citrus canker.

## Materials and Methods

### Strains, culture conditions and media

*Escherichia coli* JM109 was used for DNA subcloning and cells were cultivated at 37°C in Luria Bertani (LB) medium. Xcc (Xcc99-1330) and their derivative strains were grown at 28°C in SB, NB [33] or XVM2 [34] medium. Antibiotics were used at the following final concentrations: ampicillin (Ap) 100 μg/ml for *E*. *coli* and 25 μg/ml for Xcc, kanamycin (Km) 40 μg/ml for both strains, streptomycin (Sm), 100 μg/ml for *E*. *coli* and 50 μg/ml for Xcc, spectinomycin (Sp), 50 μg/ml for *E*. *coli* and 25 μg/ml for Xcc, tetracycline (Tc) 15 μg/ml for both strains and chloramphenicol (Cm) 30 μg/ml for *E*. *coli*.

### Strains construction

*hrpG* mutant (ΔhrpG) was constructed by marker exchange with double crossover. An 837-bp region upstream of *hrpG* (XAC1265) was amplified with the oligonucleotides 5`up EcoRI y 5`down SmaI (for oligonucleotides sequences see S1 Table) and the 1070-bp downstream region with 3`up SalI y 3`down HindIII. Using the enzymes named in the oligonucleotides, these two regions were ligated to the plasmid pK19mobGII [35]. A 2-kb DNA fragment coding for Sm/Sp resistance from pKRP13 [36] was subcloned in the SalI site of the plasmid pK19mobGII, bearing the two adjancent regions of *hrpG*. *E*. *coli* S17-1 cells transformed with this plasmid were used to perform the conjugation to Xcc and after selection for Sm/Sp resistance and Km sensitivity, ΔhrpG was verified by PCR. Complementation of ΔhrpG was done by amplification of *hrpG* with HrpGKpnIup and HrpGXhoIdown, and ligation to pBBR1MCS-3 [37] previously digested with KpnI and XhoI, rendering pBBHrpG. The mutated variant R210C was constructed similarly and the plasmid named pBBR210C. Then, plasmids were conjugated to Xcc as described above and the strains, named ΔhrpG-hrpG and ΔhrpG-R210C. These plasmids were transferred to the wild type Xcc and strains were called Xcc-hrpG and Xcc-R210C, respectively. Strains carrying *gfp* gene fusions to the *hrpG* or *hrpX* promoters were constructed as follows. Blunt-end fragments from each promoter were amplified from Xcc genomic DNA by PCR using primers phrpX up and phrpX down (for P_hrpX_) and phrpG up and phrpG down (for P_hrpG_) with Pfx50 DNA polymerase (Invitrogen). The amplified fragments were digested with HindIII and PstI restriction enzymes and cloned into pBluescript II KS+ vector previously digested with the same enzymes These plasmids were digested with HindIII and SmaI, and the resulting HindIII-SmaI fragments were subcloned into pPROBE-NT vector [38], generating pPROBE-NT-PhrpX and pPROBE-NT-PhrpG. These plasmids were introduced into Xcc and ΔhrpG as described previously.

### Expression and purification of recombinant proteins

*hrpG* and *hrpG-R210C* were amplified by PCR from pBBHrpG and pBBR210C, respectively, by using the oligonucleotides HrpGBamHIup and HrpGXhoIdown and cloned into pET32am vector that allows expression of fusion proteins to thioredoxin (Trx) and a 6X-His tag (Novagen), previously modified in our laboratory to eliminate S-tag portion and digested with the restriction enzymes BamHI and XhoI, leading to pET32-hrpG and pET32-hrpG-R210C plasmids. After transformation into *E*. *coli* BLR strain, protein synthesis was induced by 0.5 mM IPTG for 18 h at 18°C. HrpG and HrpG-R210C were purified by affinity chromatography from the soluble fraction of the bacterial lysate using a Ni-NTA agarose (Qiagen, Hilden, Germany), since the fusion protein contains a 6XHis-tag. GST-HrpG fusion protein was obtained by subcloning the BamHI-XhoI fragment from pET32-hrpG into pGEX4T3 digested with the same restriction enzymes. After transformation of BL21 (pLysS), protein synthesis was induced by 1 mM IPTG for 3 h at 28°C. GST-HrpG protein was purified by affinity chromatography from bacterial lysate using glutathione agarose matrix.

### Plant material and plant inoculations

*Citrus sinensis* cv. valencia were grown in a green house at 26 ± 2°C with a photoperiod of 16 h. Bacteria were grown in SB broth to an optical density of 1 at 600 nm, harvested by centrifugation, and resuspended in 10 mM MgCl_2_ to 10^7^ CFU/ml. Infiltrations into leaves were performed with needleless syringes. *In planta* growth assays were performed by grinding three 0.8 cm-diameter leaf discs from inoculated areas in 1 ml of 10 mM MgCl_2_, diluted, and plated to determine the CFU/cm^2^ [33]. Values are the means of three independent experiments. Results were analyzed using one-way ANOVA (p<0.05).

### RNA preparation and quantitative real-time PCR (qRT-PCR)

Total RNA from bacterial strains and from inoculated citrus leaves was extracted using TRIzol reagent (Invitrogen) at the specified times, according to the manufacturer’s instructions. After DNAse (Promega) treatment, cDNA was synthesized from 1 μg of total RNA using M-MLV RT (Promega) and the oligonucleotide dN6 (for bacteria RNA) or oligonucleotide dT (for plant RNA) was added as follows: 200 U of M-MLV RT (Promega, USA), 0.25 μg of primer dN6 and 0.5 mM of deoxynucleoside triphosphates (dNTPs) (reaction final volume: 20 μl) and incubated for 1 h at 42°C, and then for 10 min at 94°C. The qRT-PCRs were performed by combining 1 μl of cDNA template, 0.2 U of Platinum Taq DNA polymerase (Invitrogen), 1 × reaction buffer, 0.2 mM dNTPs and 20 pmol of each primer (final reaction volume, 20 μl) in a Mastercycler ep *realplex* thermal cycler (Eppendorf) using SYBR Green I (Roche) to monitor double-stranded DNA (dsDNA) synthesis. The qRT-PCR conditions were set to 95°C for 1 min, followed by 40 cycles of 95°C for 15 s, 55°C for 30 s and 72°C for 40 s. The primer pairs used for qRT-PCR are provided in S1 Table. As reference genes, a fragment of *rpoB* (XAC0965), a gene reported suitable for Xcc data normalization [39] and the actin gene for plant samples were amplified using the same qRT-PCR conditions. Values were normalized by the internal reference (Ct_r_) according to the equation ΔCt = Ct—Ct_r_, and quantified as 2^−ΔCt^ A second normalization using the control condition (Ct_c_), ΔΔCt = Ct—Ct_c_, produces a relative quantification: 2^−ΔΔCt^ [40]. Values are the means of four independent experiments. Results were analyzed using one-way ANOVA (p<0.05).

### Electrophoretic mobility shift assays (EMSA)

The DNA probe P_HrpX_ containing the *hrpX* promoter region (-414 to +30) was obtained by PCR amplification from Xcc genomic DNA with primers phrpX up and phrpX down (444 bp), and P_HrpG_ containing the *hrpG* promoter region (-412 to +48) with primers phrpG up and phrpG down (460 bp). Both regions were cloned in the vector pBluescript II KS+ previously digested with HindIII and PstI. For probes labeling, 10 pmoles of phrpX down or phrpG down were phosphorylated with 30 μCi [γ-^32^P]ATP and 20 U of T4 polynucleotide kinase. These labeled oligonucleotides were desalted with Zeba™ Spin (Thermo Scientific) and used in PCR to amplify the probes from the cloned sequences using Pfx50 DNA polymerase (Invitrogen), the reaction conditions stated above and 40 cycles of 95°C for 1 min, 50°C for 40 s, 68°C for 50 s with a final extension of 10 min at 68°C. The PCR bands were in-gel purified with “Illustra GFX” (GE Healthcare) and the purified probes were quantified using a scintillation counter. Binding was performed in 20 μl final volume of 5 mM Tris-HCl pH 7, 5% (v/v) glycerol, 25 mM KCl, 2 mM EDTA, 1 mM DTT, 1 ng (1000 cpm/μl) DNA probes and the purified proteins at the concentrations stated in the figures. The reaction mixtures were incubated for 30 min at 25°C and DNA–protein complexes were resolved by electrophoresis on a 5% (w/v) non-denaturing polyacrylamide gel in 0.5X TBE (89 mM Tris Base, 89 mM Boric acid, 2 mM EDTA), 5% (v/v) glycerol at 150 V at 4°C and were visualized and digitalized with a Storm 840 scanner (Amersham). Experiments were repeated three times with similar results.

### *In vitro* transcription assays

*In vitro* transcription assays on the promoter DNA of *hrpX* was performed. The promoter template DNA fragment of XAC1266 (P_hrpX_) was generated by PCR amplification using XccWT genomic DNA as template and primers phrpX up and phrpX-ivt down (S1 Table). The product includes the promoter region and 200 pb downstream from the annotated transcription start site [12]. For *in vitro* transcription, 16 or 32 pmoles of HrpG or HrpG-R210C were incubated for 20 min at room temperature in transcription buffer (5 mM TrisHCl pH 7, 25 mM KCl, 2.5% glycerol, 2 mM EDTA, 10 mM MgCl_2_, 1 mM DTT) containing 1 pmol of promoter template DNA. Then, 1 U of *E*. *coli* RNA polymerase Holoenzyme (sigma saturated) (Epicentre, Madison, WI, USA) was added and further incubate for 15 min at 28°C. Next, a NTP mixture (10 mM each of ATP, CTP and GTP and 50 mM [α-^32^P] UTP (3000 Ci/mmol, 10 mCi/ml)) was added to start transcription. After incubation at 28°C for 20 min, reactions were stopped by addition of loading buffer (95% formamide, 10mM NaOH, 0.05% xylene cyanol, 0.05% bromophenol blue) and incubated at 80°C for 2 min. Transcription products were run on 6% denatured polyacrylamide gel containing 7 M urea in 0,5X TBE (89 mM Tris Base, 89 mM boric acid, 2 mM EDTA) electrophoresis buffer. The transcripts obtained were visualized by autoradiography. Experiments were repeated three times with similar results.

### Pull-down assays

HrpG, HrpG-R210C or Trx (as a control) were incubated with GST-HrpG in batch conditions at room temperature for 1 h in a 1-ml reaction containing 50 mM Tris-HCl pH 7, 25 mM KCl, 1 mM DTT and 300 μl of Ni-NTA agarose. Then, the reactions were packed and washed with 25 mM Tris-HCl, 100 mM NaCl and the bound proteins were eluted with the same buffer containing 100 mM imidazole. Aliquots of 40 μl from final wash and elution samples were analyzed by 12% SDS-PAGE followed by transfer to nitrocellulose membranes and analyzed by Western Blot using anti-His and anti-GST antibodies. Experiments were repeated three times with similar results.

### Far Western assays

15 μg of purified HrpG or HrpG-R210C and Trx (as a control) were resolved by 12% SDS-PAGE and transferred to nitrocellulose membranes using 24 mM Tris, 194 mM glycine and 10% methanol buffer. The membranes were blocked with PBTS-milk (137 mM NaCl, 2.7 mM KCl, 10 mM Na_2_HPO_4_.2H_2_O, 2 mM KH_2_PO_4_ pH 7.4, supplemented with 0.05% Tween-20 and 5% powdered milk) for 1 h and then probed with GST-HrpG (50 μg/ml) in 50 mM Tris base pH 7, 25 mM KCl, 1mM DTT, 0.1% Tween-20 and 2% powdered milk buffer for 16 h at 4°C. Membranes were then washed four times with PBTS buffer and incubated with anti-GST for 1 h, then washed and incubated with anti-rabbit 1:3000. Alkaline phosphatase activity was assayed using NBT/BCIP system (Sigma). Experiments were repeated three times with similar results.

### Limited proteolysis

Purified HrpG or HrpG-R210C or a mixture of both 1:1 (5 μg) were incubated with trypsin (Sigma) (1:250, 1:125 and 1:62.5) in 25 μl of 25 mM Tris–HCl pH 8 for 30 min at room temperature. The reactions were stopped by addition of 1 mM PMSF, 2% SDS, 10% glycerol, 1% (v/v) β-mercaptoethanol and 0,01% bromophenol blue. Samples were analyzed by 15% SDS–PAGE and the bands were visualized by Coomassie stain. Experiments were repeated three times with similar results.

## Supporting Information

S1 Fig Relative expression of *hrpX* and *hrpG* in Xcc wild type and the ΔhrpG mutant. Transcriptional fusions of the promoters P_hrpX_ and P_hrpG_ to GFP reporter gene were assayed. The plasmids pPROBE-NT-PhrpX and pPROBE-NT-PhrpG were introduced into Xcc and ΔhrpG and strains were grown in XVM2. Values of fluorescence obtained for the mutant were expressed relative to the values obtained for the wild type Xcc. The experiment was repeated 3 times with three technical replicates each. Error bars indicate the standard deviation.
(PDF)

S2 Fig HrpG wild type and the R210C mutant bind similarly to *hrpG* promoter. Electrophoretic mobility shift assay of ^32^P-labeled *hrpG* promoter and purified HrpG and HrpG-R210C. Numbers in the top of the lanes indicate pmoles of protein added to the assay.
(PDF)

S3 Fig The αCTD fragment of RNA polymerase does not cause a supershift in the HrpG-P_hrpX_ complex. Supershift assay using HrpG and HrpG-R210C pre-incubated with the αCTD fragment of RNA polymerase and then with the P_hrpX_ promoter region. Lane 1 shows the binding of 16 pmoles of of HrpG to P_hrpX_ as control. Lanes 2 and 3: P_hrpX_ was incubated with 50 and 100 pmoles of αCTD, respectively and lanes 4 and 5 shows the pre-incubation of HrpG (lane 4) and HrpG-R210C (lane 5) with αCTD (50 pmoles) and then incubation with P_hrpX_.
(PDF)

S1 Table List of all the oligonucleotides used in this study. (PDF)
