# Supplementary material for: The Amino Acid Arginine 210 of the Response Regulator HrpG of Xanthomonas citri subsp. citri Is Required for HrpG Function in Virulence
Source: PLoS One. 2015 May 11;10(5):e0125516. doi: 10.1371/journal.pone.0125516 (PMC4427454; doi:10.1371/journal.pone.0125516)
Supplement: S1 Fig — Transcriptional fusions of the promoters PhrpX and PhrpG to GFP reporter gene were assayed. The plasmids pPROBE-NT-PhrpX and pPROBE-NT-PhrpG were introduced into Xcc and ΔhrpG and strains were grown in XVM2. Values of fluorescence obtained for the mutant were expressed relative to the values obtained for the wild type Xcc. The experiment was repeated 3 times with three technical replicates each. Error bars indicate the standard deviation. (PDF) [file pone.0125516.s001.pdf]

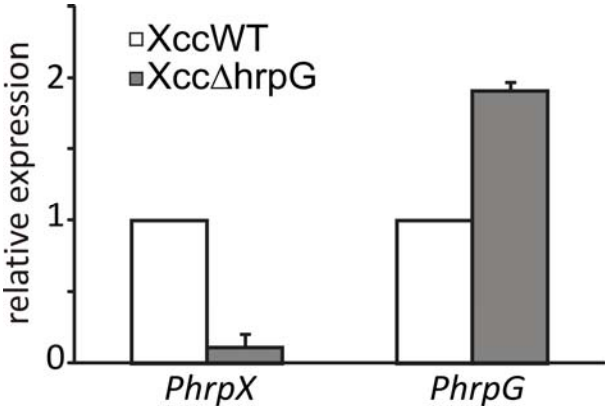

S1 Figure. Relative expression of *hrpX* and *hrpG* in Xcc wild type and the ΔhrpG mutant. Transcriptional fusions of the promoters P_{hrpX} and P_{hrpG} to GFP reporter gene were assayed. The plasmids pPROBE-NT-PhrpX and pPROBE-NT-PhrpG were introduced into Xcc and ΔhrpG and strains were grown in XVM2. Values of fluorescence obtained for the mutant were expressed relative to the values obtained for the wild type Xcc. The experiment was repeated 3 times with three technical replicates each. Error bars indicate the standard deviation.
