# Supplementary material for: The Amino Acid Arginine 210 of the Response Regulator HrpG of Xanthomonas citri subsp. citri Is Required for HrpG Function in Virulence
Source: PLoS One. 2015 May 11;10(5):e0125516. doi: 10.1371/journal.pone.0125516 (PMC4427454; doi:10.1371/journal.pone.0125516)
Supplement: S2 Fig — Electrophoretic mobility shift assay of 32P-labeled hrpG promoter and purified HrpG and HrpG-R210C. Numbers in the top of the lanes indicate pmoles of protein added to the assay. (PDF) [file pone.0125516.s002.pdf]

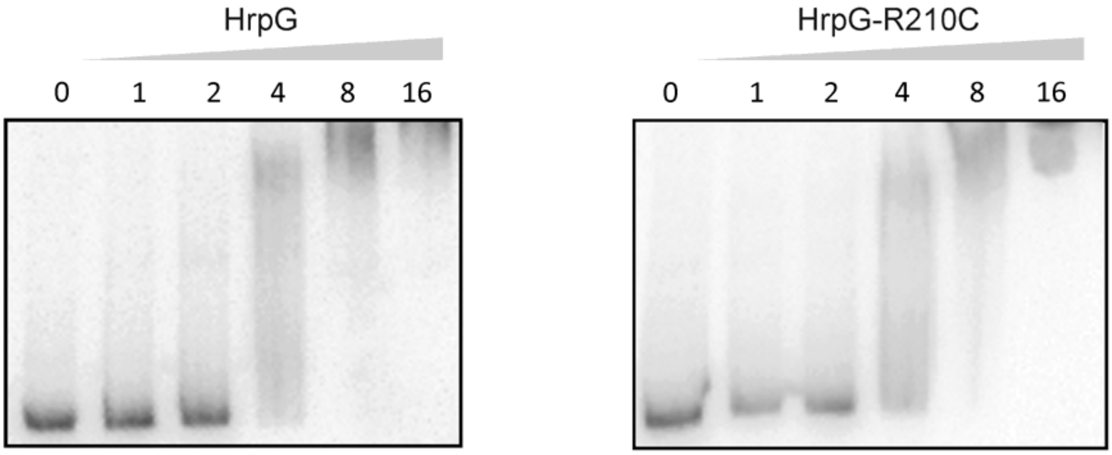

S2 Figure. HrpG wild type and the R210C mutant bind similarly to *hrpG* promoter. Electrophoretic mobility shift assay of ^{32}P-labeled *hrpG* promoter and purified HrpG and HrpG-R210C. Numbers in the top of the lanes indicate pmoles of protein added to the assay.
