# Supplementary material for: The Amino Acid Arginine 210 of the Response Regulator HrpG of Xanthomonas citri subsp. citri Is Required for HrpG Function in Virulence
Source: PLoS One. 2015 May 11;10(5):e0125516. doi: 10.1371/journal.pone.0125516 (PMC4427454; doi:10.1371/journal.pone.0125516)
Supplement: S3 Fig — Supershift assay using HrpG and HrpG-R210C pre-incubated with the αCTD fragment of RNA polymerase and then with the PhrpX promoter region. Lane 1 shows the binding of 16 pmoles of of HrpG to PhrpX as control. Lanes 2 and 3: PhrpX was incubated with 50 and 100 pmoles of αCTD, respectively and lanes 4 and 5 shows the pre-incubation of HrpG (lane 4) and HrpG-R210C (lane 5) with αCTD (50 pmoles) and then incubation with PhrpX. (PDF) [file pone.0125516.s003.pdf]

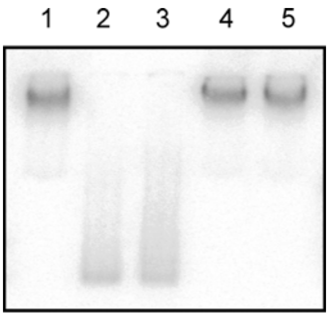

S3 Figure. The αCTD fragment of RNA polymerase does not cause a supershift in the HrpG-P_{hrpX} complex. Supershift assay using HrpG and HrpG-R210C pre-incubated with the αCTD fragment of RNA polymerase and then with the P_{hrpX} promoter region. Lane 1 shows the binding of 16 pmoles of of HrpG to P_{hrpX} as control. Lanes 2 and 3: P_{hrpX} was incubated with 50 and 100 pmoles of αCTD, respectively and lanes 4 and 5 shows the pre-incubation of HrpG (lane 4) and HrpG-R210C (lane 5) with αCTD (50 pmoles) and then incubation with P_{hrpX}.
