# Supplementary material for: The Amino Acid Arginine 210 of the Response Regulator HrpG of Xanthomonas citri subsp. citri Is Required for HrpG Function in Virulence
Source: PLoS One. 2015 May 11;10(5):e0125516. doi: 10.1371/journal.pone.0125516 (PMC4427454; doi:10.1371/journal.pone.0125516)
Supplement: S1 Table — (PDF) [file pone.0125516.s004.pdf]

S1 Table. List of all the oligonucleotides used in this study.

**Name**	**Oligonucleotide sequence**	**Use**
5`up EcoRI	GATC**GAATTC**CCGGTCTCTCTCTTTGGG	Mutant construction
5`down SmaI	AGCT**CCCGGG**GAGTGGTCGTTCATTTAGG	Mutant construction
3’up SalI	GCAT**GTCGAC**CGATACGGAGGCAACGAC	Mutant construction
3’down HindIII	TAGC**AAGCTT**CAGACACCGCTCACTGCC	Mutant construction
HrpGKpnIup	GACT**GGTACC**GTGCATGAACGACCACTCTCCC	Mutant complementation
HrpGBamHIup	GACT**GGATCC**ATGAACGACCACTCTCCC	Protein expression
HrpGXhoIdown	GCAT**CTCGAG**ATCAAACACCCTTCGCTTTAA	Mutant complementation/ Protein expression
HrpX-RT up	CGATGATGAGGTCAGTTTGT	qRT-PCR
HrpX-RT down	ACTGCGCAAAGCAATTCAAC	qRT-PCR
HrcC-RT up	TTCGTCTGGTACTACGATGG	qRT-PCR
HrcC-RT down	CCGAAACGGTATCCACATAC	qRT-PCR
hrpB2-RT up	AACCAAGCGCTTGTGAATCG	qRT-PCR
hrpB2-RT down	CTATTGGTTCTTGACCAGTG	qRT-PCR
RpoB up	ACGAGAAGGAATTCGAAGCA	qRT-PCR
RpoB down	GTTGGAGACCACACCCTTGT	qRT-PCR
CsLOB1 up	TCCACCAACCGAACCATACA	qRT-PCR
CsLOB1 down	GGCACTTGCTTCATAGACCAT	qRT-PCR
Actin up	TCAATTGGATACTTCAAAGTCAAAAT	qRT-PCR
Actin down	ACGTGAATTCTAGTGTTTCGATAAGT	qRT-PCR
phrpX up	GCATAAGCTTCAGTGATCTGCCCTCGTGTC	Promoter cloning<br>In vitro transcription
phrpX down	GCATCTGCAGGATCGCTGCAAAGTAAGTGG	Promoter cloning
phrpG up	GCATAAGCTTTGCCGACCTATTCCAGTCAC	Promoter cloning
phrpG down	GCATCTGCAGCGTCAACAGGAACACCGATC	Promoter cloning
phrpX-ivt down	CAGCGCCCTGTCCGTCGCG	In vitro transcription
